# Translating evidence to patient care through caregivers: a systematic review of caregiver-mediated interventions

**DOI:** 10.1186/s12916-018-1097-4

**Published:** 2018-07-12

**Authors:** Kirsten M. Fiest, Christiane Job McIntosh, Danielle Demiantschuk, Jeanna Parsons Leigh, Henry T. Stelfox

**Affiliations:** 1 Departments of Critical Care Medicine and Community Health Sciences, O’Brien Institute for Public Health and Hotchkiss Brain Institute, Cumming School of Medicine, University of Calgary, 2500 University Drive NW, Calgary, AB T2N 1N4 Canada; 2 Department of Critical Care Medicine, Cumming School of Medicine, University of Calgary, 2500 University Drive NW, Calgary, AB T2N 1N4 Canada; 3 Department of Critical Care Medicine, O’Brien Institute for Public Health, Cumming School of Medicine, University of Calgary, 2500 University Drive NW, Calgary, AB T2N 1N4 Canada; 4 Departments of Critical Care Medicine, Medicine and Community Health Sciences, O’Brien Institute for Public Health, Cumming School of Medicine, University of Calgary, 2500 University Drive NW, Calgary, AB T2N 1N4 Canada

**Keywords:** Systematic review, Caregiver-mediated, Quality improvement, Knowledge translation, Implementation science, Intervention, Review, Translational medical research

## Abstract

**Background:**

Caregivers may promote the uptake of science into patient care and the practice of evidence-informed medicine. The purpose of this study was to determine whether caregiver-mediated (non-clinical caregiver-delivered) interventions are effective in improving patient, caregiver, provider, or health system outcomes.

**Methods:**

We searched the MEDLINE, Embase, PsycINFO, Cumulative Index of Nursing and Allied Health, and Scopus databases from inception to February 27, 2017. Interventions (with a comparison group) reporting on a quality improvement intervention mediated by a caregiver and directed to a patient, in all ages and patient-care settings, were selected for inclusion. A three-category framework was developed to characterize caregiver-mediated interventions: inform (e.g., provide knowledge), activate (e.g., prompt action), and collaborate (e.g., lead to interaction between caregivers and other groups [e.g., care providers]).

**Results:**

Fifty-six studies met the inclusion criteria, and 64% were randomized controlled trials (RCTs). The most commonly assessed outcomes were patient- (*n* = 40) and caregiver-oriented (*n* = 33); few health system- (*n* = 10) and provider-oriented (*n* = 2) outcomes were reported. Patient outcomes (e.g., satisfaction) were most improved by caregiver-mediated interventions that provided condition and treatment education (e.g., symptom management information) and practical condition-management support (e.g., practicing medication protocol). Caregiver outcomes (e.g., stress-related/psychiatric outcomes) were most improved by interventions that activated caregiver roles (e.g., monitoring blood glucose) and provided information related to that action (e.g., why and how to monitor). The risk of bias was generally high, and the overall quality of the evidence was low-moderate, based on Grading of Recommendations Assessment Development and Evaluation ratings.

**Conclusions:**

There is a large body of research, including many RCTs, to support the use of caregiver-mediated interventions that inform and activate caregivers to improve patient and caregiver outcomes. Select caregiver-mediated interventions improve patient (*inform-activate*) and caregiver (*inform-activate-collaborate*) outcomes and should be considered by all researchers implementing patient- and family-oriented research.

**Systematic review:**

PROSPERO, CRD42016052509.

**Electronic supplementary material:**

The online version of this article (10.1186/s12916-018-1097-4) contains supplementary material, which is available to authorized users.

## Background

Patient- and family-centered care (PFCC) research, where patients and families partner with providers in the planning and delivery of healthcare, represents a shift from solely studying provider-identified priorities [1, 2]. PFCC represents a cultural change in healthcare that acknowledges that patients and their caregivers are central figures in decision-making and the delivery of care [1, 2]. Perceptions of PFCC and its benefits are high among patients and family members, though there are barriers to its implementation, including a purported lack of provider knowledge of PFCC, system barriers (e.g., lack of personnel, visiting hours), and a perceived increase in provider burden [3–5]. The uptake of PFCC interventions is limited by these barriers, and the science of PFCC is lagging behind scientific, clinical, operational, and public interest and desire.

PFCC may provide a mechanism for quality improvement initiatives designed to implement evidence-informed practices into patient care. Patients and caregivers can act as agents of change, making evidence more accessible and providing a method for implementing science into patient care, regardless of the clinical setting [2]. Due to the nature of their illness and the use of invasive therapies, some patients may not be able to act as active agents of change [6]; children, and a growing number of adults with complex care needs, represent populations who require a caregiver. This presents a unique role for caregivers to be engaged in PFCC, though the evidence supporting the delivery and effectiveness of caregiver-mediated interventions is limited. Because of the growing role of patients and caregivers in care, there is an opportunity to engage and empower them to promote the uptake of science into patient care and the practice of evidence-informed medicine.

In order to effectively carry out PFCC, we must create, implement, and measure the effect of PFCC interventions. A recent systematic review of patient-mediated interventions suggested benefit [7]. There is a large and diverse literature on caregiver-mediated interventions, though no systematic review has been conducted. We therefore asked the question: Are caregiver-mediated interventions (interventions delivered in some part by the caregiver) effective in improving patient, caregiver, provider, or health system outcomes?

## Methods

This systematic review was conducted and reported per the Preferred Reporting Items for Systematic Reviews and Meta-Analyses (PRISMA) criteria [8] (see Additional file 1 for the checklist). An a priori protocol was published online through PROSPERO (registration number CRD42016052509; Additional file 2). Ethical approval was not required, as previously published data were used.

### Patient and stakeholder involvement

Our methods were informed by our stakeholders: a 2-day stakeholder (patients, families, researchers, care providers) engagement meeting was held (November 17–18, 2016, Calgary, Canada) to determine relevant, patient-oriented priorities. Attendees included three former intensive care unit (ICU) patients, four family members of former ICU patients, 19 researchers, and 10 care providers. Patients and family members were asked to identify relevant and important patient-oriented priorities in critical care medicine; the discussion was open and guided by an experienced facilitator. Study findings will be presented to these patients and family members at the next stakeholder engagement meeting planned for the Fall of 2018.

### Outcome measures

Based on feedback from stakeholders, the primary research question was: Are caregiver-mediated interventions effective in improving patient, caregiver, provider, or health system outcomes? Secondary research questions were the following: (1) What types of interventions have been deemed effective? (2) Does effectiveness vary according to the intervention type, patient population, caregiver group, contextual factors, or implementation strategy? (3) How have these interventions been evaluated?

### Populations, interventions, comparators, settings, and study designs

Inclusion criteria for studies were as follows: (1) interventional study design with a comparison group; (2) reporting on an intervention *mediated* by caregivers and directed to a patient; (3) conducted in a patient-care setting of any age group; and (4) in any language, and published at any time. Studies were excluded if they were not interventional in design, reported on interventions *directed* at the caregiver, or were not conducted in the context of patient care (e.g., took place at home). For the purposes of this review, we established definitions for key concepts and defined: (1) a caregiver as any non-clinical person who regularly provides support to the patient and is in some way directly implicated in the patient’s care or directly affected by the patient’s health problem; (2) caregiver-mediated as the caregiver being directly involved in delivering the intervention; (3) a patient as someone who has a health condition, is at risk for developing a health condition, or is attending a patient-care setting to receive care; and (4) a patient-care setting as any setting where a patient may receive care for an existing health condition or preventative care from a healthcare professional. We distinguish caregiver*-mediated* interventions from caregiver*-directed* interventions—where the caregiver is the target of the intervention and is not involved in delivering the intervention him/herself. Quality improvement (or implementation science or knowledge translation) was defined as the synthesis, dissemination, exchange, and application of health-related knowledge and research [9]. Studies were excluded if they were only abstracts or were not primary research.

### Data sources and searches

The search strategy (Additional file 3) was developed with the assistance of a medical librarian (H.G.) and in consultation with experts in quality improvement in healthcare (H.T.S., J.P.L.). The MEDLINE (OVID), Embase (OVID), PsycINFO (OVID), Cumulative Index of Nursing and Allied Health (CINAHL [EBSCO]), and Scopus (Elsevier) bibliographic databases were searched from inception to February 27, 2017. Using subject headings, keywords, and synonyms, we searched the following domains: (1) caregivers; (2) intervention; (3) mediated; (4) quality improvement/knowledge translation; (5) clinical care. The reference lists of included papers were reviewed to identify potential studies missed in the search.

### Study selection

All titles and abstracts were reviewed independently in duplicate by two reviewers (K.M.F., C.J.M.); any study selected by either reviewer at this stage was selected to move on to the next stage. The full text of all articles was reviewed independently in duplicate by two of three reviewers (K.M.F., C.J.M., D.D.); all articles selected by both reviewers at this stage were included in the final review. Disagreements were resolved by discussion or the involvement of a third reviewer when necessary. References were managed in EndNote X7 (Clarivate Analytics, Philadelphia, PA, USA).

### Data extraction and quality assessment

Two reviewers (C.J.M., D.D.) abstracted data independently, in duplicate, using a data collection sheet developed and piloted by the study team. Information on study design, study characteristics (e.g., age, setting), patient population (e.g., infants, older adults; as defined by the individual papers), caregiver group (e.g., spouses, parents, family caregivers [which may include parents when undefined]), intervention, and control group was abstracted, and risk of bias and strength of the evidence (see below) assessed. Translation was sought for all non-English articles (*n* = 7; 2 French, 2 Cantonese, 1 Spanish, 1 Turkish, 1 Serbo-Croatian).

The Cochrane Collaboration’s risk of bias tool was used to evaluate the internal validity of all included studies [10]. To be assigned an overall low risk of bias, all domains needed to be rated as low. The presence of a single high or unclear item resulted in the overall risk of bias being rated as high or unclear, respectively. The strength of the evidence was described as low, moderate, or high according to the Grading of Recommendations Assessment Development and Evaluation (GRADE) working group approach [11].

### Data synthesis and analysis

Findings were summarized using descriptive statistics ( median, frequencies) and their accompanying measures of variability ( interquartile range [IQR]) where appropriate using STATA 14.1. Meta-analysis was not possible due to heterogeneity in study design, type of intervention, and outcomes reported. Using a framework of engagement described in previous research on patient-mediated interventions [7] and adapted from a previous meta-review [12], we categorized interventions into three engagement strategies: *inform* ( interventions that provide knowledge), *activate* ( interventions that prompt action), and *collaborate* ( interventions that lead to interaction between the caregiver and other groups such as providers or other caregivers) (Additional file 1). Within these three strategies were 13 types of engagement support, including lifestyle advice, psychological strategies, and information on available resources (examples in Additional file 1). Interventions were further classified as single-component ( involving a single component/engagement support) or complex interventions ( involving more than one component/engagement support) [13]. Study outcomes were classified as patient-oriented, caregiver-oriented, provider-oriented, or health system-oriented.

### Role of the funding source

The study funder had no role in the study design; in the collection, analysis, or interpretation of data; in the writing of the report; or in the decision to submit the article for publication. The researchers are independent from the study funders.

## Results

### Results of the search

**Fig. 1:**
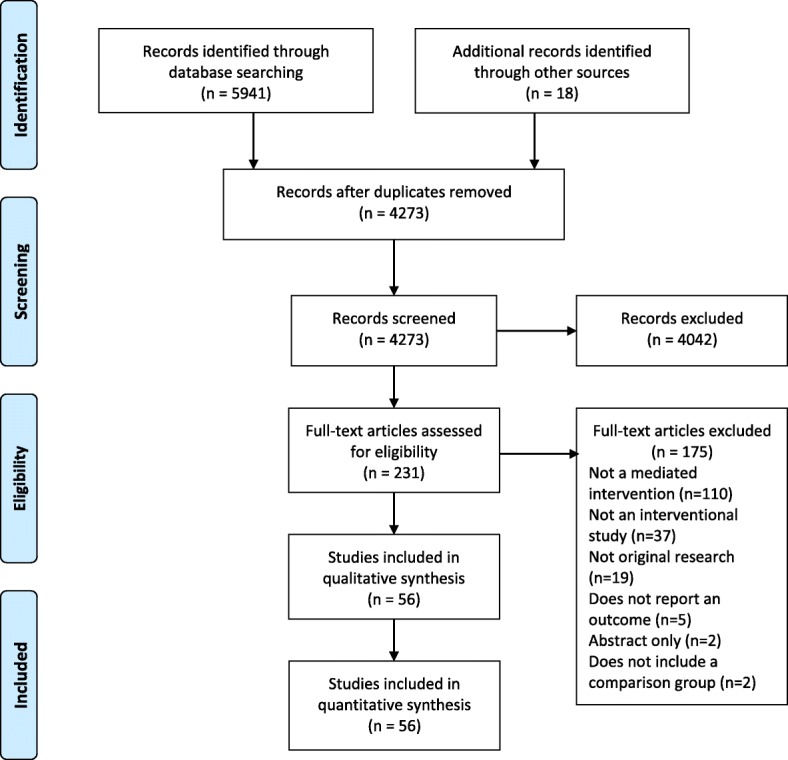
Study flow diagram

We screened 4273 unique abstracts and reviewed 231 full-text articles; 175 full-text articles were excluded, the most common reasons being that the intervention was not mediated by a caregiver 110/175) or that the study did not include an intervention (37/175) (Fig. 1). Hand searching resulted in the inclusion of 18 additional studies. Characteristics of the 56 studies meeting all inclusion criteria are described in Additional file 2.

### Description of included studies

The 56 intervention studies [1, 2, 14–67] were published between 1984 and 2017 and conducted in the USA (*n* = 32, 57.1%), Canada (*n* = 3, 5.4%), and 14 other countries (*n* = 1 or 2 studies each) (Additional file 2). Most studies (*n* = 36, 64.3%) were randomized controlled trials [14, 15, 18, 20, 24, 26–31, 33–36, 38, 39, 41–47, 50, 51, 54–59, 62, 64–66] and conducted in outpatient settings (including primary care and the community) (*n* = 33). Caregivers were most commonly parents (*n* = 25, 44.6%) [14–18, 20, 25, 28, 30, 34, 36, 37, 39–41, 46–48, 49, 53, 58, 60, 64–66] and family caregivers more broadly—family caregivers could include parents, but the term was broadly defined—(*n* = 17, 30.4%) [1, 2, 22, 23, 31–33, 35, 42, 44, 45, 54, 56, 59, 61, 63, 67], with the most common populations receiving care being premature/low birth weight infants (*n* = 8, 14.3%) [14, 17, 18, 40, 47, 49, 65, 66], children with asthma (*n* = 6, 10.7%) [26, 27, 34, 41, 46, 56], and stroke survivors (*n* = 4, 7.0%) [33, 45, 51, 55]. More than half of the interventions (*n* = 30, 53.6%) were directed towards children, with the interventions mediated by parents or family caregivers [14–18, 20, 22, 24–28, 30, 34, 36, 37, 39–41, 46–49, 53, 56, 58, 60, 64–66].

### Interventions and assessments

**Fig. 2:**
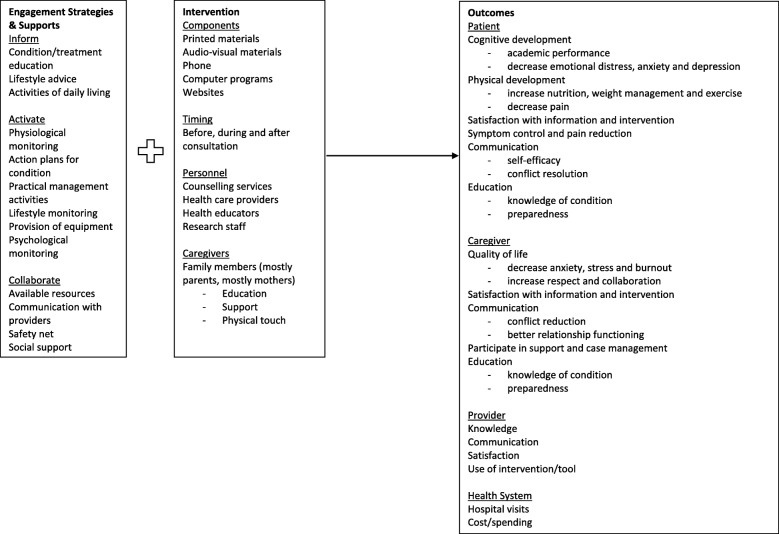
Conceptual framework derived from synthesis of data

Caregiver-mediated interventions were categorized by the type of engagement strategy and support provided (Additional file 1). Figure 2 provides a conceptual framework of the engagement strategy and supports, caregiver-mediated interventions employed, and reported outcomes. All interventions were considered complex, with a median of 4 (IQR 2–6) engagement supports. The most common type of engagement strategy was *inform* (*n* = 55, 98.2%), followed by *activate* (*n* = 54, 96.4%), and *collaborate* (*n* = 27, 48.2%). The most common engagement support used as part of the *inform* strategies was condition/treatment education (*n* = 47, 83.9%), for the *activate* strategies the most common engagement support was practical management activities (*n* = 40, 71.4%), and for the *collaborate* strategies the most common engagement support was providing a safety net to caregivers (*n* = 11, 19.6%). All *inform* engagement strategies were accompanied by either *activate* and/or *collaborate* strategies. The most common pairing of engagement strategies was *inform*-*activate* (*n* = 28, 50.0%), and among those, educational engagement supports combined with practical management strategies (*n* = 8). For example, one *inform*-*activate* engagement strategy included family caregiver education and involvement in the basic care of patients admitted to the ICU; specifically, caregivers were provided with information on the advantages of their involvement in the basic care of the patient and details on the activities they could carry out, including general hygiene, mouth hygiene, mobilization, and feeding [42]. Similarly, two *inform-collaborate* engagement strategies included an education program aimed to improve knowledge about stroke and stroke prevention (*inform*), described services available in hospital and the community (*collaborate*), and provided an opportunity to ask questions and receive group support (*collaborate*) [51].

### Outcomes

Patient- and caregiver-oriented outcomes were most commonly reported (*n* = 40 and *n* = 33, respectively) (Table 1). Health system-oriented outcomes were reported in 10 studies and provider-oriented outcomes in two studies. Patient-oriented outcomes included cognitive development, symptom control, and communication. Caregiver-oriented outcomes included anxiety, depression, and satisfaction. Provider-oriented outcomes included improved communication with caregivers and satisfaction with the intervention. Health system-oriented outcomes included length of hospital stay, number of hospital visits, and costs. Interventions with condition and treatment education (*inform*) and practical management (*activate*) engagement supports were generally effective in improving patient-reported outcomes. For caregiver-reported outcomes, engagement supports that included action plans (*activate*), practical management (*activate*), or lifestyle monitoring (*activate*), and condition and treatment education (*inform*) or lifestyle advice (*inform*) engagement supports were generally effective in improving outcomes. Provider outcomes were improved by providing caregivers engagement supports related to activities of daily living (*inform*) and notice of available resources (*collaborate*). Interventions with social support and safety net engagement supports (*collaborate*) tended to improve health system outcomes. No studies reported whether harmful effects were associated with the interventions.

**Table 1:** Type and effectiveness of interventions for primary study outcomes

Type of engagement	Patient outcomes	Caregiver outcomes	Provider outcomes	Health system outcomes
*Inform-activate-collaborate*	5⇑ 9⇔	11⇑ 4⇔	–	5⇑ 3⇔
*Inform-activate*	18⇑ 5⇔	9⇑ 5⇔	1⇑	1⇑ 1⇔
*Inform-collaborate*	1⇔	2⇑	1⇑	–
*Activate-collaborate*	1⇑ 1⇔	2⇔	–	–

Numbers represent the number of studies reporting on each outcome

⇑ statistically significant positive effect of intervention, ⇔ no statistically significant effect of intervention or mixed effect of intervention, ⇓ statistically significant harmful effect of intervention

### Location of patient care

Interventions conducted in inpatient (or combined inpatient and outpatient) settings (*n* = 34) had more statistically significant positive effects on patient and caregiver outcomes than no/mixed effects (Table 2). In outpatient settings (including primary care and the community) (*n* = 39), interventions examining caregiver outcomes had more statistically significant positive effects than no/mixed effects. The same trend was observed for patient outcomes. Most inpatient interventions (*n* = 15, 44.1%) were *inform-activate*, while most outpatient interventions (*n* = 18, 46.2%) were *inform-activate-collaborate.*

**Table 2:** Type and effectiveness of interventions for select subgroup analyses

Study population	Patient outcomes	Caregiver outcomes
All	24⇑ 16⇔	22⇑ 11⇔
Location of care		
Inpatient	11⇑ 7⇔	10⇑ 6⇔
Outpatient	13⇑ 9⇔	12⇑ 5⇔
Patient population		
Infants/children/adolescents	14⇑12⇔	7⇑7⇔
Adults	7⇑1⇔	6⇑1⇔
Older adults	3⇑3⇔	9⇑3⇔
GRADE criteria		
Very low-low	8⇑ 9⇔	7⇑ 6⇔
Moderate-high	16⇑7⇔	15⇑ 5⇔

Numbers represent the number of studies reporting on each outcome

⇑ statistically significant positive effect of intervention, ⇔ no statistically significant effect of intervention or mixed effect of intervention, ⇓: statistically significant harmful effect of intervention

### Patient population

The majority of interventions were directed towards children (newborn–18 years) (*n* = 30, 53.6%), followed by adults (greater than 18 years) (*n* = 11, 19.6%) and older adults (greater than 55 years) (*n* = 15, 26.8%). Ten pediatric studies focused on newborn, low birth weight, or premature infants, while the remainder (*n* = 20) reported on children overall. In studies of infants, mothers (*n* = 6) delivered the intervention more frequently than both parents (*n* = 4), while in studies of children, both parents (*n* = 15) or a caregiver (*n* = 5) delivered the intervention. In studies of adults, the most commonly studied populations were persons with stroke (*n* = 3), persons with comorbid substance misuse, and persons in the ICU (*n* = 2). Among adult populations, the persons providing care were caregivers broadly (not specifically defined) (*n* = 10) or spouses (*n* = 1). Engagement strategies for adults were either *inform-activate* (*n* = 6) or *inform-activate-collaborate* (*n* = 5)*.* Interventions directed at older adults were most commonly conducted in persons with dementia or Alzheimer’s disease (*n* = 8) and utilized an *inform-activate-collaborate* engagement strategy (*n* = 11). Caregiver outcomes were reported in more studies of older adults (*n* = 13) than patient (*n* = 8), health system (*n* = 4), and provider (*n* = 1) outcomes. For both patient and caregiver outcomes in infants, children, or adolescents, there was a similar number of positive effects and mixed effects (Table 2). In adults, there were considerably more positive effects observed for both patient and caregiver outcomes. In older adults, caregiver outcomes were more frequently improved, though this was not observed for patient outcomes.

### Acceptability and feasibility

The acceptability and feasibility of caregiver-mediated interventions were inconsistently reported in the included studies, with less than half (*n* = 26, 46.4%) reporting response rates or process evaluations.

### Risk of bias assessment and strength of evidence

None of the 56 included studies had a low overall risk of bias; risk of bias was high in 47 studies (83.9%) and unclear in nine studies (16.1%) (Additional file 4). The overall strength of the evidence based on GRADE criteria was low-moderate (Additional file 5). A lack of blinding of participants (i.e., caregivers and patients) and outcome assessors (i.e., patients not blinded to patient reported outcomes) was the main factor that limited the strength of the evidence.

When the analyses were restricted to studies with moderate or greater strength of evidence (*n* = 33, 58.9%), the results were similar, with most statistically significant effects observed for *inform*-*activate* engagement strategies for patient outcomes (Additional file 6).

## Discussion

We utilized systematic review methodology to synthesize the literature on the effectiveness of caregiver-mediated interventions. There is a large and recent body of literature (including many randomized controlled trials [RCTs]) suggesting that complex interventions comprised of *inform-activate* and *inform-activate*-*collaborate* engagement strategies are associated with improved patient and caregiver outcomes. In contrast, there is very little literature regarding provider and health system outcomes, though all interventions involved care providers and/or the health system in some way. The potential for unintended consequences or harm from caregiver-mediated interventions was not explored in the included studies, and much of the literature focused on the parent-child relationship.

That interventions with an *inform* engagement strategy always included parts of *activate* and/or *collaborate* engagement strategies is noteworthy, as research suggests that education-only interventions (*inform* in this language) in various settings and populations are some of the weakest [68–71]. Education with prompts for action or education with active collaboration may circumvent the limitations of education alone [68–71]. Most interventions were mediated by parents or family caregivers (any family member, though not defined specifically), though there was heterogeneity in both the type of caregiver included and in how caregiver was defined. It is important to recognize that one caregiver is not the same as another; they may differ in their commitment, readiness, and capacity to fulfill the role. Validated tools to assess caregiver readiness (Willingness to Care Scale [72], Caregiver Self Expectations Inventory [73]) should be employed alongside caregiver-mediated interventions in this context [74]. Interventions to support caregivers once they are already in a caregiving role dominate the literature [75–77], with little or no information supporting the use of interventions to prepare the caregiver prior to entering the caregiving role. Nearly all studies of adults found improvement in both patient and caregiver outcomes, though in pediatric studies only half found improvement in patient and caregiver outcomes. Improved outcomes may be dependent on whether the caregiver-patient relationship is parent-child (i.e., parents providing support to children) versus child-parent (i.e., children providing support to parents).

All included interventions were complex (including multiple components). Thus, we are uncertain of the ”active ingredients” of the interventions and how these components affect outcomes. The knowledge translation literature suggests that complex interventions may be no better than single-component interventions [68, 69]. If this holds true for caregiver-mediated interventions, then single-component interventions may represent a resource-efficient means of improving patient, caregiver, provider, and health system outcomes. We observed variation in reported outcomes according to the types of engagement pairings, suggesting that different combinations of interventions might have differing effects: the previously observed effect of simple-component versus complex interventions may not hold true for caregiver-mediated interventions. *Inform*-*activate* engagement strategies provide the strongest evidence for improving patient outcomes through caregiver-mediated interventions*.* Based on the results of the current study, we recommend interventions that provide both condition-specific information and prompts or tools to support action for managing a condition to the caregiver. For example, an intervention to encourage early mobilization of patients recovering from a serious illness might include both information about how the illness affects the patient’s conditioning (*inform*) paired with instructions and prompts for caregiver-directed bedside physical therapy (*activate*). It is unclear how these interventions will apply in other contexts, though they appear to work in children and adults with any family caregiver in an inpatient setting. There was a trend towards more positive than equivocal effects for improved caregiver outcomes in an outpatient setting. *Inform*-*activate*-*collaborate* engagement strategies are supported by the strongest evidence to improve caregiver outcomes. We recommend utilizing this type of strategy for infants or children in outpatient or community settings with any family caregiver. For example, there could be a three-component (*inform-activate-collaborate*) telephone coaching intervention for parents of children with asthma (*activate*) combined with lifestyle advice on how to avoid asthma triggers (*inform*) and the provision of contact information for extra information or clinical supports (*collaborate*). These interventions have the potential to improve caregiver outcomes. At this time, there have been few evaluations of interventions using *inform-collaborate* (e.g., providing information to support activities of daily living and a list of available resources) or *activate*-*collaborate* (e.g., creating action plans specific to a medical condition and the provision of contact information for extra support) engagement strategies. For interventions conducted in the community (either partially or fully), it appears that *collaborate* engagement supports are necessary to improve outcomes.

Despite the large body of literature, there are a number of key gaps that must be addressed: provider and health system outcomes, the role of caregiver, the potential harms of caregiver-mediated interventions; and the economic impact of these interventions. Only two studies explored provider outcomes of caregiver-mediated interventions. Providers represent an important mechanism for implementing PFCC research [4, 78, 79]; it is essential to measure the impact that caregiver-mediated interventions have on this population. Ten studies reported on health system outcomes, which included hospital and emergency room visits and associated costs. As all caregiver-mediated interventions engage with the healthcare system, it is important to measure the impact they have on these limited resources. Within our aging population, investigations should explore whether the inverse effect of the parent-child dyad applies to caregiver-mediated interventions—that is, when children become the caregiver, are the same outcomes observed? The evidence from parent-child relationships may not inform the child-parent caregiver relationship in this context. No included studies reported on potential harms of caregiver-mediated interventions (e.g., falls among patients being mobilized by caregivers, caregiver feelings of guilt related to intervention adherence, conflict with providers); unintended effects of these interventions are an important area that requires further study. Caregiver-mediated interventions represent an opportunity for healthcare cost savings by transferring select care responsibilities from the clinical team to the caregiver. Future studies should assess the financial impact of employing caregiver-mediated interventions.

This study has a number of strengths, including the use of rigorous methodology defined by an a priori published protocol and adherence to recommended reporting criteria. We utilized previously validated tools to assess both the quality and strength of the evidence presented. The search was executed in five bibliographic databases, it was not restricted by language, and it was intentionally broad to ensure that caregiver-mediated interventions across all areas of healthcare were included. By focusing on healthcare settings, home-based interventions were excluded, including many studies of caregiver-mediated interventions in persons with dementia. In spite of these strengths, there are limitations to note. Our findings are limited by the methodological quality of the included studies; the risk of bias was generally high, and the overall quality of the evidence was low-moderate. When restricted to studies of at least moderate quality, there was a trend towards observing more positive effects. Risk of bias tends to be high in studies where blinding does not occur, as is the case in many of these interventions, and does not necessarily mean they are methodologically poor [80]. The literature is rich with RCTs, the quality of which is limited by methodological factors inherent in this type of research (e.g., lack of blinding) that are unlikely to be overcome in the future. We did not search the gray literature and could have missed studies, though our comprehensive search strategy included databases indexing both North American and European journals and full-text hand-searching was completed. We noted a lack of scientific abstracts, limited use of applicable keywords, and variable terminology to describe caregiver-mediated interventions in the included studies. This highlights the importance of full-text hand-searching, as these papers may not be found through standard database searching. All included papers reported on complex interventions, and thus it is not possible to determine which parts of the interventions were effective. The lack of effect in some studies may be a failure of implementation (few process evaluations conducted to inform) or a failure of measurement (i.e., use of the wrong measure) and not the intervention itself.

## Conclusions

There is a large body of research, including many RCTs, to support the use of caregiver-mediated interventions that inform and activate caregivers to improve patient and caregiver outcomes. Additional study is needed to evaluate provider and health system outcomes, interventions for relationships outside of the parent-child role, potential harms, and economic implications. We recommend consideration of *inform-activate* and *inform-activate-collaborate* interventions when implementing patient and family-oriented research.

## Additional files

Additional file 1: Characteristics of Caregiver-Mediated Interventions. Description of the engagement strategies and engagement supports and examples of each utilized in the study. (DOCX 17 kb)

Additional file 2: Characteristics of Included Studies. Table of the studies included in the systematic review with information including setting, participant population, intervention, and outcomes. (DOCX 120 kb)

Additional file 3: PRISMA Checklist. A completed PRISMA checklist for study transparency. (DOCX 24 kb)

Additional file 4: Risk of Bias Assessment. Risk of bias ratings for each included study. (DOCX 20 kb)

Additional file 5: GRADE Ratings. GRADE ratings for each included study. (DOCX 19 kb)

Additional file 6: Type and Effectiveness of Interventions for Primary Study Outcomes in Studies with Moderate or High Strength Evidence According to the GRADE Criteria. Main results restricted to those studies with a moderate or high strength of evidence according to the GRADE criteria. (DOCX 13 kb)

## Abbreviations

GRADE: Grading of Recommendations Assessment Development and Evaluation
IQR: Interquartile range
PFCC: Patient and family-centered care
PRISMA: Preferred Reporting Items for Systematic Reviews and Meta-Analyses
RCT: Randomized controlled trial
