# Supplementary material for: Translating evidence to patient care through caregivers: a systematic review of caregiver-mediated interventions
Source: BMC Med. 2018 Jul 12;16:105. doi: 10.1186/s12916-018-1097-4 (PMC6042352; doi:10.1186/s12916-018-1097-4)
Supplement: Supplementary file 1 — Characteristics of Caregiver-Mediated Interventions. Description of the engagement strategies and engagement supports and examples of each utilized in the study. (DOCX 17 kb) [file 12916_2018_1097_MOESM1_ESM.docx]

Additional file 1. Characteristics of caregiver-mediated knowledge translation interventions^*^

| Type of Engagement | Type of Engagement Support | Examples of Intervention |
| --- | --- | --- |
| **Inform**  Information that provides caregivers with knowledge about their family member’s condition and an understanding of how to participate in managing it | Condition and treatment education | Information and education about the condition, prognosis, what to expect and its management (e.g., caregivers learn about asthma management through text, phone and in-person coaching) |
|  | Lifestyle advice | Information and guidance on lifestyle behaviours that support disease management, including home safety and identifying triggers for relapse (e.g., family support for those patients managing substance abuse and psychosis) |
|  | Activities of daily living | Information and advice for caregivers on how to help patients undertake activities of daily living such as hygiene, dressing, preparing meals and transportation (e.g., caregivers supported to ensure adequate nutrition for patient) |
| **Activate**  Prompts or tools to prompt action for patients in actively managing the condition and enhancing quality of life for both patients and caregivers | Physiological monitoring | Evaluation tools to log and monitor physiological measures for assessment and to share with clinicians (e.g., regular monitoring of blood sugar in persons with diabetes) |
|  | Action plans for condition | Guidance for both patients and caregivers specific to medical condition, providing signs of worsening condition, how to adjust treatment and respond if deterioration continues (e.g,. a care-plan developed by practitioners for caregivers of patients with Alzheimer’s to follow) |
|  | Practical management activities | Practicing, reviewing, or delivering condition management activities (e.g., a telephone coaching intervention for parents of children with asthma) |
|  | Lifestyle monitoring | Prompts for caregivers to support adherence to recommended lifestyle behaviours (e.g., caregivers of low-birth-weight children monitor their weight) |
|  | Provision of equipment | Provision of equipment necessary to manage a condition (e.g., a magnifying glass for skin examinations for melanoma) |
|  | Psychological strategies | Mechanisms for problem-solving, goal-setting, reframing and relaxation (e.g., monitoring speech development and social behaviors in children with autism) |
| **Collaborate**  Interventions that lead to interaction and engagement | Available resources | Contact details for organizations that offer information, psycho-social support or financial aid and assistance with appropriate referrals (e.g., referrals to community supports for patients and caregivers dealing with anorexia nervosa) |
|  | Communication with providers | Guidance and prompts to facilitate communication with healthcare professionals (e.g., a nurse who meets with parents of very low birth-weight infants) |
|  | Safety netting | Provision of contact information for extra information or support, or increased visits if necessary (e.g., contact information for a home care program) |
|  | Social support | Programs that offer support, mentoring or socializing for both caregivers and patients (e.g., caregivers participated in group support sessions for patients with dementia) |

^*^Adapted from Gagliardi(7) & Taylor(12)
