# Supplementary material for: Translating evidence to patient care through caregivers: a systematic review of caregiver-mediated interventions
Source: BMC Med. 2018 Jul 12;16:105. doi: 10.1186/s12916-018-1097-4 (PMC6042352; doi:10.1186/s12916-018-1097-4)
Supplement: Supplementary file 2 — Characteristics of Included Studies. Table of the studies included in the systematic review with information including setting, participant population, intervention, and outcomes. (DOCX 120 kb) [file 12916_2018_1097_MOESM2_ESM.docx]

Additional file 2. Characteristics of Included Studies^*^

| **Author (Year)** | **Setting** | **Study design** | **Participant Population** | **Caregivers** | **Intervention** | **Control** | **Outcomes** |
| --- | --- | --- | --- | --- | --- | --- | --- |
| Bao (2005)^1^ | Inpatient | Controlled Trial | Premature infants | Parents | Education program and skills training | Standard care | No statistically significant differences |
| Barrera (1986)^2^ | Community | Three-arm randomized controlled trial | Preterm infants <2000 g | Parents | Parent-infant or developmental programming | Standard care | No statistically significant differences |
| Melnyk (2006)^3^ | Inpatient | Randomized Control Trial | Low birth weight premature infants | Parents | Creating Opportunities for Parent Empowerment (COPE) | Information regarding hospital services and policies | Mothers had significantly less stress in NICU and less depression and anxiety at 2 months (p<0.05). General stress was not affected (p=0.25). Infants had shorter NICU (p=0.05) and hospital length of stay (p=0.02) |
| Anderson (1999)^4^ | Outpatient | Three-arm randomized controlled trial | Children with Type 1 diabetes | Parents | (1) Teamwork or (2) Attention Control | Standard care | No statistically significant differences |
| Dawson (2014)^5^ | Outpatient and Primary Care | Two-phase randomized controlled trial | Children with BMI >85th percentile | Parents | Weight feedback via motivational interviewing | Best practice care | Parents increased concern over their child’s weight after intervention (p=0.03) |
| Tyler (2016)^6^ | Inpatient | Randomized Control Trial | Children with BMI >95^th^ percentile | Parents | Information and 7 clinic visits | Information and 4 clinic visits | Connecting families to providers in managing child’s weight |
| Golley (2006)^7^ | Community | Randomized Control Trial | Children at risk of becoming overweight | Parents | Parenting skills and/or education | Standard care | Non-significant reduction in BMI between intervention and control groups |
| Fiks (2015)^8^ | Primary Care | Randomized Control Trial | Children with moderate to severe persistent asthma | Parents | MyAsthma, an EHR-linked patient portal | Standard care | Intervention families had fewer: emergency department visits, received more controller medications, days of school and work missed (p=0.001) |
| Garbutt (2010)^9^ | Community | Randomized Control Trial | Children with asthma | Parents | Telephone coaching program | Standard care | Quality of life was most significant among parents who received 9+ calls (p=0.04) |
| Horn (2014)^10^ | Outpatient | Single blind, parallel groups, randomized controlled trial | Children with asthma | Parents | Parent Empowerment Program for Asthma Care | Standard care | No statistically significant differences |
| Nelson (2011)^11^ | Outpatient | Randomized Control Trial | Children with asthma | Parents | Asthma coaching | Standard care | Reduction of Emergency Department visits for children with asthma |
| Helgadottir (2014)^12^ | Outpatient and Community | Randomized Control Trial | Children who had a tonsillectomy | Parents | Pain management education with distraction | Pain management education | Intervention group had decreased pain behavior on post-operative day 1 (p<0.001) |
| Ozonoff (1998)^13^ | Outpatient and Community | Controlled Trial | Children with autism | Parents | Treatment and Education of Autistic and related Communication-impaired Children | Standard care | Significant improvement for children in treatment group: Imitation subset (p<0.05), Fine motor subtest (p<0.05) and cognitive performance (p<0.06) |
| Sénéchal (2013)^14^ | Outpatient | Pre-post study with waitlist | Children with autism | Parents | Behavioral intervention/communication | Waitlist control | No statistically significant results on child or maternal behaviours |
| Bagner (2013)^15^ | Outpatient | Quasi-experimental | Children with developmental delay | Parents | Parent-Child Interaction Therapy | N/A | Children from Involved-father families have most improved outcomes |
| Brown (2015)^16^ | Community | Multiple-baseline single-case intervention studies | Children with Down syndrome, Autism Spectrum Disorder, or Developmental Delays | Parents | Parent-implemented communication - KidTalk-Tactics Project | N/A | Parent-implemented communication improves child’s communication and parent’s responsiveness |
| Sun (2016)^17^ | Inpatient | Open-label randomized controlled trial | Children with mental illness who are violent to caregiver | Parents | Child-parent Focused Violence Program | Standard Care | Reduction of violence to caregiver |
| Gomez (2014)^18^ | Primary Care | Pre-post study | Youth with externalizing behavior problems | Parents | Parent Management Training with Behavioral Health Consultants | N/A | Following treatment, significant Improvement occurred in global distress (p<0.001); satisfaction with health services (0.49) |
| Achenbach (1993)^19^ | Inpatient and Community | Three-arm randomized controlled trial | Low birth weight infants | Mothers | Mother-Infant Transaction Program | Standard care | Slight improvement cognitive development (p<0.10) |
| Beeghly (1995)^20^ | Inpatient and Community | Randomized Control Trial | Newborn infants | Mothers | Braselton Neonatal Assessment Scale and Interview | In-depth Interview | No statistically significant differences |
| Holditch-Davis (2014)^21^ | Inpatient | Three-arm randomized trial | Infants who weighed <1750g at birth | Mothers | (1) Auditory-Tactile-Visual-Vestibular or (2) Kangaroo Care | Standard care | Improvement in maternal psychological distress (p<0.05) |
| Melnyk (2001)^22^ | Inpatient | Randomized Control Trial | Low birth weight premature infants | Mothers | Creating Opportunities for Parent Empowerment (COPE) | Audiotaped information | Infants had significantly higher mental development scores at 3 (p=0.05) and 6 (p<0.05) months corrected age |
| Nurcombe (1984)^23^ | Inpatient | Randomized Control Trial | Low birth weight infants | Mothers | Mother-Infant Transaction Program | Standard care | Maternal self-confidence, role satisfaction, and attitude improved in the intervention group (all p< 0.021). No differences were found on infant cognitive outcomes |
| Pridham (2006)^24^ | Inpatient and Community | Randomized Trial | Very low birth weight infants (<1500g) | Mothers | Guided participation and guidelines | Standard care and guidelines | No statistically significant differences |
| Melnyk (2004)^25^ | Inpatient | Randomized Control Trial | Children in a pediatric intensive care unit | Mothers | Creating Opportunities for Parent Empowerment (COPE) | Educational-behavioral intervention | No differences in maternal state anxiety and total negative mood state during or after hospitalization. No differences in PTSD symptoms. Children had fewer behavioral symptoms (p<0.05) |
| Berthelsen (2016)^26^ | Inpatient | Pre-post study | Older adults with arthritis and total hip replacement | Spouse | Case management | Standard care | Significant improvement in activities of daily living-function for patients (p=0.014). No statistical differences for the spouses |
| Eloniemi-Sulkava (2009)^27^ | Community | Randomized Control Trial | Older adults with Dementia | Spouse | Support of care coordinator, support groups and information | Standard care | Intervention group less likely to be in long-term institutional care (p=0.03). Intervention led to a significant reduction in the use of community services and costs (p=0.03) |
| Mittelman (1996)^28^ | Outpatient | Randomized Control Trial | Adults with Alzheimer Disease | Spouse | Family counselling and support groups | Standard care | Intervention patients increased delay to nursing home (p=0.02) |
| Robinson (2007)^29^ | Outpatient | Randomized Control Trial | Adults at risk of melanoma | Partner | Partner-learning skin checking | Solo-learning skin checking | Program effects of partner assisted intervention – behavior and psychological – improved (p<0.001) |
| Blauw-Hospers (2011)^30^ | Inpatient and Community | Randomized Trial | Infants at high risk for developmental disorders | Family caregiver | Coping with and Caring for Infants with Special Needs | Traditional Infant Physical Therapy | Neurological conditions, Alberta Infant Motor Scale scores, Mental Development Index scores were not significantly different |
| Watson (2009)^31^ | Outpatient | Randomized Control Trial | Children with asthma | Family caregiver | Asthma education program | Standard care | Reduction in emergency department visits (p=0.0004). Improved caregiver quality of life (p=0.04) |
| Souaibi (2016)^32^ | Inpatient | Controlled trial | Adults with schizophrenia | Family caregiver | Teacher Education Program for Families of Patients with schizophrenia | Standard Care | Aide caregiver in dealing with needs of patients suffering from schizophrenia |
| Tantirangsee (2015)^33^ | Outpatient | Randomized Control Trial | Adults with Schizophrenia/other psychoses & Substance Use Disorders | Family caregiver | Family support (brief intervention/with family support) | Advice only | No statistically significant differences |
| Mueser (2013)^34^ | Community | Randomized Control Trial | Adults with co-occurring substance use and severe psychiatric disorders | Family caregiver | Family education and training in communication and problem-solving skills | Family education program | Decrease in opiate use (p=0.05) |
| Garcia-Huidobro (2001)^35^ | Primary Care and Community | Randomized Control Trial | Adults with type 2 diabetes mellitus | Family caregiver | Family oriented intervention to improve metabolic control | Standard care | Significant reduction in HbA1c (p<0.001). |
| Martinez (2003)^36^ | Inpatient | Randomized Control Trial | Intensive care unit patients | Family caregiver | Education and involvement in basic care | Standard care | Family satisfaction increased (p=0.026). Nurses improved relationship with family (p=0.021) |
| Mitchell (2009)^37^ | Inpatient | Non-equivalent control group pre-post | Intensive care unit patients | Family caregiver | Nursing-led family involvement in care | Standard care | Patients saw increase in perceived respect; collaboration; support; family-centered care. (p<0.001) |
| Skoog (2016)^38^ | Inpatient | Pre-post study | Cardiothoracic intensive care unit patients post-surgery | Family caregiver | Facilitated sense-making | Not applicable | Significant reduction in caregiver state anxiety (p=0.001) |
| Galvin (2011)^39^ | Inpatient | Randomized Control Trial | Adults with acute stroke | Family caregiver | Family-mediated exercise therapy | Routine physical therapy | Statistically significant improvement in walking activity (p<0.05). Caregivers reported less strain (p<0.01) |
| Nayeri (2014)^40^ | Inpatient | Post-test only randomized controlled trial | Adults who have survived a stroke | Family caregiver | Family-centered care program | Standard care | Significant increase across therapeutic regime (adherence to rehabilitation, medication, and dietary regimens) (p<0.001) |
| Li (2003)^41^ | Inpatient | Randomized clinical trial | Hospitalized older adults | Family caregiver | Creating Avenues for Relative Empowerment | Standard care | Caregiver increased confidence in implementing strategies for elderly patients in hospital (p=0.06); increased role rewards (p=0.04) |
| Toye (2016)^42^ | Community | Single blind randomized controlled trial | Older adults discharged from hospital | Family caregiver | Further Enabling Care at Home Program | Standard care | Caregiver felt prepared and supported in giving care |
| Boltz (2014)^43^ | Inpatient | Comparative trial with repeated measures | Older adults | Family caregiver | Family-centered Function-focused Care | Standard care | Significant increase in preparedness for caregiving (p=0.007); decrease in depression (p=0.0001); decrease in anxiety (p=0.0001) |
| Fortinsky (2002)^44^ | Primary Care and Community | Pre-post study | Older adults with dementia | Family caregiver | Alzheimer's Service Coordination Program | N/A | Caregiver self-efficacy improved (p=0.016); caregiver and physicians were satisfied with the intervention |
| Fortinsky (2009)^45^ | Primary Care and Community | Randomized Control Trial | Older adults with dementia | Family caregiver | Dementia care consultants - counseling, support and education | Education only | Improvement for caregiver outcomes: symptom management self-efficacy (p=0.89); depression score (p=0.41) |
| Gitlin (2006)^46^ | Community | Quasi-experimental | Adults with dementia | Family primary caregiver | Adult Day Services (ADS) Plus program | Standard care | Participants reported less depression (p=0.016) and greater well-being (p=-.001) at 3-months and had fewer nursing home placements. |
| Boudreau (2013)^47^ | Primary Care | Randomized waitlist controlled trial | Latino children with BMI >85th percentile | Caregiver | Power Up classes and coaching | 6 month delay | Slight changes in BMI z-scores (p=0.31); lipid panel (p=0.08); and moderate-vigorous physical activity (p=0.88) |
| Butz (2005)^48^ | Outpatient and Community | Randomized Control Trial | Children with asthma | Caregiver | Support of a nurse/health educator, home visits and education | Standard care | Caregiver asthma communication educational intervention had no impact on decreasing symptoms |
| Chen (2013)^49^ | Outpatient | Randomized Control Trial | Children with asthma | Caregiver | Self-management interactive support | Standard care | Significant improvement in attitude regarding asthma management (p<0.05); forced expiratory value (p<0.05); emergency room visits (p<0.05) |
| Grover (2011)^50^ | Community | Randomized Control Trial | Adults with Anorexia Nervosa | Caregiver | Overcoming Anorexia Online | Beat support | Caregiver distress was reduced through web-based support intervention |
| Bass (2013)^51^ | Outpatient and Community | Matched intervention and comparison sites | Veterans with dementia | Caregiver | Partners in Dementia Care program | Standard care | Care program significantly improved caregiver outcomes: unmet needs, strain, depression and support resources |
| Mavandadi (2017)^52^ | Community | Randomized Control Trial | Veterans with dementia | Caregiver | Telephone-based collaborative management program | General materials and standard care | Reduction of caregiver distress and improved ability to cope |
| Callahan (2006)^53^ | Primary Care | Randomized Control Trial | Older adults with Alzheimer Disease | Caregiver | Interdisciplinary care management | Augmented standard care | Patients had fewer behavioral and psychological symptoms of dementia at 18 months (p=0.01). Caregivers had less distress (p=0.03) and improvement in depression symptoms (p=0.02) at 12 and 18 months, respectively |
| Wang (2015)^54^ | Community | Single blind randomized controlled trial | Stroke survivors | Caregiver | Caregiver-mediated home-based physical therapy intervention | Standard care | Intervention improved strength (p=0.002) mobility (p<0.001) recovery domain (p<0.001) |
| Rodgers (1999)^55^ | Inpatient | Randomized Control Trial | Older adults who have survived a stroke | Caregiver | Stroke Education Program | Standard care | Improvement for carers in social functioning (p=0.04). Increased knowledge (patients p=0.02; carers p=0.01). Increased satisfaction with information (p=0.004) |
| Rodriguez-Gonzalo (2015)^56^ | Inpatient | Cluster Randomized Trial | Older adults | Primary Informal Caregivers | Intensive educational program | Generic Speech | Improvement in in hygiene caregiving (p=0.0001) |

*Sorted by caregiver type, then participant population. Family caregiver may include any family member. Caregiver may include any family members or non-family members.

N/A: not applicable; CI: confidence interval

1. Bao XLNCRGfLIoCPoPItE, Intervention. [Lowering incidence of cerebral palsy of premature infants through early intervention]. *Zhonghua Er Ke Za Zhi.* 2005;43(4):244-247.

2. Barrera ME, Rosenbaum PL, Cunningham CE. Early home intervention with low-birth-weight infants and their parents. *Child development.* 1986;57(1):20-33.

3. Melnyk BM, Feinstein NF, Alpert-Gillis L, et al. Reducing premature infants' length of stay and improving parents' mental health outcomes with the Creating Opportunities for Parent Empowerment (COPE) neonatal intensive care unit program: a randomized, controlled trial. *Pediatrics.* 2006;118(5):e1414-1427.

4. Anderson BJB, J.; Ho, J.; Laffel, L. M. B. An office-based intervention to maintain parent-adolescent teamwork in diabetes management: impact on parent involvement, family conflict, and subsequent glycemic control. *Diabetes Care.* 1999;22(5):713-721.

5. Dawson AMB, D. A.; Cox, A.; Williams, S. M.; Treacy, L.; Haszard, J.; Meredith-Jones, K.; Hargreaves, E.; Taylor, B. J.; Ross, J.; Taylor, R. W. Using motivational interviewing for weight feedback to parents of young children. *J Paediatr Child Health.* 2014;50(6):461-470.

6. Tyler DO, Horner SD. A primary care intervention to improve weight in obese children: A feasibility study. *Journal of the American Association of Nurse Practitioners.* 2016;28(2):98-106.

7. Golley RK, Magarey AM, Baur LA, Steinbeck KS, Daniels LA. Twelve-month effectiveness of a parent-led, family-focused weight-management program for prepubertal children: a randomized, controlled trial. *Pediatrics.* 2007;119(3):517-525.

8. Fiks AGM, S. L.; Karavite, D. J.; Suh, A.; O'Hara, R.; Localio, A. R.; Ross, M.; Grundmeier, R. W. Parent-reported outcomes of a shared decision-making portal in asthma: a practice-based RCT. *Pediatrics.* 2015;135(4):e965-973.

9. Garbutt JM, Banister C, Highstein G, et al. Telephone coaching for parents of children with asthma: impact and lessons learned. *Arch Pediatr Adolesc Med.* 2010;164(7):625-630.

10. Horn IBM, S. J.; Gillespie, C. W.; Burke, K. M.; Godoy, L.; Teach, S. J. Randomized trial of a health communication intervention for parents of children with asthma. *J Asthma.* 2014;51(9):989-995.

11. Nelson KAH, G. R.; Garbutt, J.; Trinkaus, K.; Fisher, E. B.; Smith, S. R.; Strunk, R. C. A randomized controlled trial of parental asthma coaching to improve outcomes among urban minority children. *Arch Pediatr Adolesc Med.* 2011;165(6):520-526.

12. Helgadottir HLW, M. E. A randomized controlled trial of the effectiveness of educating parents about distraction to decrease postoperative pain in children at home after tonsillectomy. *Pain Manag Nurs.* 2014;15(3):632-640.

13. Ozonoff S, Cathcart K. Effectiveness of a home program intervention for young children with autism. *Journal of autism and developmental disorders.* 1998;28(1):25-32.

14. Sénéchal CL, S.; Thermidor, G. Parents as co-therapists: A winning solution for treating autistic children. *Ann. Med.-Psychol.* 2013;171(9):603-609.

15. Bagner DM. Father's role in parent training for children with developmental delay. *J Fam Psychol.* 2013;27(4):650-657.

16. Brown JAW, Juliann J. Effects of a Triadic Parent-Implemented Home-Based Communication Intervention for Toddlers. *Journal of Early Intervention.* 2015;37(1):44-68.

17. Sun G-C, Hsu M-C. Effects of nurse-led child- and parent-focused violence intervention on mentally ill adult patients and victimized parents: A randomized controlled trial. *International Journal of Nursing Studies.* 2016;60:79-90.

18. Gomez DB, Ana J.; Andrews, Arthur R., III; Cavell, Timothy A.; Pastrana, Freddie A.; Gregus, Samantha J.; Ojeda, Carlos A. Delivering parent management training in an integrated primary care setting: Description and preliminary outcome data. *Cognitive and Behavioral Practice.* 2014;21(3):296-309.

19. Achenbach TMH, C. T.; Aoki, M. F.; Rauh, V. A. Nine-year outcome of the Vermont intervention program for low birth weight infants. *Pediatrics.* 1993;91(1):45-55.

20. Beeghly MB, T. Berry; Flannery, Kathleen A.; Nugent, J. Kevin; Barrett, David E.; Tronick, Edward Z. Specificity of preventative pediatric intervention effects in early infancy. *Journal of Developmental and Behavioral Pediatrics.* 1995;16(3):158-166.

21. Holditch-Davis DW-T, R. C.; Levy, J. A.; O'Shea, T. M.; Geraldo, V.; David, R. J. Maternally administered interventions for preterm infants in the NICU: Effects on maternal psychological distress and mother-infant relationship. *Infant Behav. Dev.* 2014;37(4):695-710.

22. Melnyk BM, Alpert-Gillis L, Feinstein NF, et al. Improving cognitive development of low-birth-weight premature infants with the COPE program: a pilot study of the benefit of early NICU intervention with mothers. *Research in nursing & health.* 2001;24(5):373-389.

23. Nurcombe B, Howell DC, Rauh VA, Teti DM, Ruoff P, Brennan J. An intervention program for mothers of low-birthweight infants: preliminary results. *Journal of the American Academy of Child Psychiatry.* 1984;23(3):319-325.

24. Pridham KAK, Mary M.; Limbo, Rana K.; Paradowski, Jill; Rudd, Nancy; Meurer, John R.; Uttech, Ann; Henriques, Jeffrey B. Guiding Mothers' Management of Health Problems of Very Low Birth-Weight Infants. *Public Health Nursing.* 2006;23(3):205-215.

25. Melnyk BM, Alpert-Gillis L, Feinstein NF, et al. Creating opportunities for parent empowerment: program effects on the mental health/coping outcomes of critically ill young children and their mothers. *Pediatrics.* 2004;113(6):e597-607.

26. Berthelsen CBK, J. The SICAM-trial: evaluating the effect of spouses' involvement through case management in older patients' fast-track programmes during and after total hip replacement. *J Adv Nurs.* 2016;4:4.

27. Eloniemi-Sulkava U, Saarenheimo M, Laakkonen ML, et al. Family care as collaboration: effectiveness of a multicomponent support program for elderly couples with dementia. Randomized controlled intervention study. *J Am Geriatr Soc.* 2009;57(12):2200-2208.

28. Mittelman MS, Ferris SH, Shulman E, Steinberg G, Levin B. A family intervention to delay nursing home placement of patients with Alzheimer disease. A randomized controlled trial. *Jama.* 1996;276(21):1725-1731.

29. Robinson JKT, R.; Stapleton, J. Examination of mediating variables in a partner assistance intervention designed to increase performance of skin self-examination. *J Am Acad Dermatol.* 2007;56(3):391-397.

30. Blauw-Hospers CHD, Tineke; Hulshof, Lily J.; Bos, Arend F.; Hadders-Algra, Mijna. Pediatric Physical Therapy in Infancy: From Nightmare to Dream? A Two-Arm Randomized Trial. *Physical Therapy.* 2011;91(9):1323-1338.

31. Watson WT, Gillespie C, Thomas N, et al. Small-group, interactive education and the effect on asthma control by children and their families. *CMAJ : Canadian Medical Association journal = journal de l'Association medicale canadienne.* 2009;181(5):257-263.

32. Souaibi L, Choueifati D, Kerbage H, Richa S. Therapeutic education of families of patients with schizophrenia: The Lebanese Hôtel-Dieu de France model. *Ann. Med.-Psychol.* 2016;174(8):677-682.

33. Tantirangsee NA, S.; Marsden, J. Effects of a brief intervention for substance use on tobacco smoking and family relationship functioning in schizophrenia and related psychoses: A randomised controlled trial. *J. Subst. Abuse Treat.* 2015;51:30-37.

34. Mueser KT, Glynn SM, Cather C, et al. A randomized controlled trial of family intervention for co-occurring substance use and severe psychiatric disorders. *Schizophrenia bulletin.* 2013;39(3):658-672.

35. Garcia-Huidobro DB, M.; Brahm, P.; Puschel, K. Family intervention to control type 2 diabetes: a controlled clinical trial. *Fam Pract.* 2011;28(1):4-11.

36. Martínez MCRM, F. R.; Del Pino, A. R. C.; Almenara, M. I. M.; Bannik, J. T.; Caballero, L. J. F.; Macías, G. C.; Bernet, L. A.; González, C. A. Family involvement in the critically ill patient basic care. *Enfermería Intensiva.* 2003;14(3):96-108.

37. Mitchell M, Chaboyer W, Burmeister E, Foster M. Positive effects of a nursing intervention on family-centered care in adult critical care. *American Journal of Critical Care.* 2009;18(6):543-552.

38. Skoog M, Milner KA, Gatti-Petito J, Dintyala K. The Impact of Family Engagement on Anxiety Levels in a Cardiothoracic Intensive Care Unit. *Crit Care Nurse.* 2016;36(2):84-89.

39. Galvin RC, T.; O'Grady, E.; Murphy, B.; Stokes, E. Family mediated exercise intervention [fame]: Evaluation of a novel form of exercise delivery after stroke. *Physiotherapy (United Kingdom).* 2011;97:eS387-eS388.

40. Nayeri NDM, Sepideh; Razi, Shadan Pedram; Kazemnejad, Anoushirvan. Investigating the effects of a family-centered care program on stroke patients' adherence to their therapeutic regimens. *Contemporary Nurse: A Journal for the Australian Nursing Profession.* 2014;47(1/2):88-96.

41. Li H, Melnyk BM, McCann R, et al. Creating avenues for relative empowerment (CARE): a pilot test of an intervention to improve outcomes of hospitalized elders and family caregivers. *Research in nursing & health.* 2003;26(4):284-299.

42. Toye C, Parsons R, Slatyer S, et al. Outcomes for family carers of a nurse-delivered hospital discharge intervention for older people (the Further Enabling Care at Home Program): Single blind randomised controlled trial. *International Journal of Nursing Studies.* 2016;64:32-41.

43. Boltz MR, B.; Chippendale, T.; Galvin, J. Testing a family-centered intervention to promote functional and cognitive recovery in hospitalized older adults. *J Am Geriatr Soc.* 2014;62(12):2398-2407.

44. Fortinsky RHU, Christine G.; Garcia, Ramon I. Helping family caregivers by linking primary care physicians with community-based dementia care services: The Alzheimer's Service Coordination Program. *Dementia: The International Journal of Social Research and Practice.* 2002;1(2):227-240.

45. Fortinsky RHK, M.; Kleppinger, A.; Kenyon-Pesce, L. Dementia care consultation for family caregivers: collaborative model linking an Alzheimer's association chapter with primary care physicians. *Aging Ment Health.* 2009;13(2):162-170.

46. Gitlin LN, Reever K, Dennis MP, Mathieu E, Hauck WW. Enhancing quality of life of families who use adult day services: Short- and long-term effects of the adult day services plus program. *Gerontologist.* 2006;46(5):630-639.

47. Boudreau ADAK, D. S.; Gonzalez, W. I.; Dimond, M. A.; Oreskovic, N. M. Latino families, primary care, and childhood obesity: A randomized controlled trial. *American Journal of Preventive Medicine.* 2013;44(3 SUPPL. 3):S247-S257.

48. Butz A, Kub J, Donithan M, et al. Influence of caregiver and provider communication on symptom days and medication use for inner-city children with asthma. *J Asthma.* 2010;47(4):478-485.

49. Chen SHH, J. L.; Yeh, K. W.; Tsai, Y. F. Interactive support interventions for caregivers of asthmatic children. *J Asthma.* 2013;50(6):649-657.

50. Grover MN, U.; Mohammad-Dar, L.; Glennon, D.; Ringwood, S.; Eisler, I.; Williams, C.; Treasure, J.; Schmidt, U. A randomized controlled trial of an Internet-based cognitive-behavioural skills package for carers of people with anorexia nervosa. *Psychological Medicine.* 2011;41(12):2581-2591.

51. Bass DMJ, K. S.; Snow, A. L.; Wilson, N. L.; Morgan, R.; Looman, W. J.; McCarthy, C. A.; Maslow, K.; Moye, J. A.; Randazzo, R.; Garcia-Maldonado, M.; Elbein, R.; Odenheimer, G.; Kunik, M. E. Caregiver outcomes of partners in dementia care: effect of a care coordination program for veterans with dementia and their family members and friends. *J Am Geriatr Soc.* 2013;61(8):1377-1386.

52. Mavandadi S, Wright EM, Graydon MM, Oslin DW, Wray LO. A randomized pilot trial of a telephone-based collaborative care management program for caregivers of individuals with dementia. *Psychological Services.* 2017;14(1):102-111.

53. Callahan CM, Boustani MA, Unverzagt FW, et al. Effectiveness of collaborative care for older adults with Alzheimer disease in primary care: a randomized controlled trial. *Jama.* 2006;295(18):2148-2157.

54. Wang T-CT, Alan C.; Wang, Jiun-Yi; Lin, Yu-Te; Lin, Ko-Long; Chen, Jiun Jiang; Lin, Bei Yi; Lin, Tai Ching. Caregiver-mediated intervention can improve physical functional recovery of patients with chronic stroke: A randomized controlled trial. *Neurorehabilitation and Neural Repair.* 2015;29(1):3-12.

55. Rodgers H, Atkinson C, Bond S, Suddes M, Dobson R, Curless R. Randomized controlled trial of a comprehensive stroke education program for patients and caregivers. *Stroke.* 1999;30(12):2585-2591.

56. Rodríguez-Gonzalo AG-M, Carlos; Ocaña-Colorado, Ascensión; Baquera-De Micheo, M. José; Morel-Fernández, Silvia. Efficiency of an intensive educational program for informal caregivers of hospitalized, dependent patients: cluster randomized trial. *BMC Nursing.* 2015;14(1):1-12.
