# Supplementary material for: Translating evidence to patient care through caregivers: a systematic review of caregiver-mediated interventions
Source: BMC Med. 2018 Jul 12;16:105. doi: 10.1186/s12916-018-1097-4 (PMC6042352; doi:10.1186/s12916-018-1097-4)
Supplement: Supplementary file 4 — Risk of Bias Assessment. Risk of bias ratings for each included study. (DOCX 20 kb) [file 12916_2018_1097_MOESM4_ESM.docx]

Additional file 4. Risk of Bias Assessment

| **Author** | **ROB1:** random sequence generation | **ROB2:** allocation concealment | **ROB3:**  blinding of participants/  personnel | **ROB4:** blinding of outcome assessors | **ROB5:** incomplete outcome assessment | **ROB6:** other sources of bias | **ROB7:** selective reporting | **ROB overall** |
| --- | --- | --- | --- | --- | --- | --- | --- | --- |
| Bao (2005) | High | High | High | High | Unclear | Unclear | Unclear | **High** |
| Barrera (1986) | Low | Unclear | High | High | Unclear | Unclear | Unclear | **High** |
| Melnyk (2006) | Unclear | Low | Low | Low | Unclear | Low | Unclear | **Unclear** |
| Anderson (1999) | High | High | High | High | Unclear | Unclear | Low | **High** |
| Dawson (2014) | Low | Low | Low | High | Low | Low | Unclear | **High** |
| Tyler (2016) | High | High | High | High | Low | Unclear | Unclear | **High** |
| Golley (2006) | High | Low | Low | Low | Low | Unclear | Unclear | **High** |
| Fiks (2015) | Low | Low | Low | Unclear | Low | Low | Unclear | **Unclear** |
| Garbutt (2010) | Low | Low | High | High | Low | Unclear | Unclear | **High** |
| Horn (2014) | Low | Low | Unclear | Unclear | High | Low | High | **High** |
| Nelson (2011) | High | High | Unclear | Unclear | Unclear | High | Unclear | **High** |
| Helgadottir (2014) | Low | Unclear | High | High | Low | Low | Unclear | **High** |
| Ozonoff (1998) | High | High | High | Low | Unclear | unclear | Low | **High** |
| Sénéchal (2013) | High | High | High | High | Unclear | Unclear | Unclear | **High** |
| Bagner (2013) | High | High | High | High | Unclear | Unclear | High | **High** |
| Brown (2015) | High | High | High | High | Unclear | Unclear | Unclear | **High** |
| Sun (2016) | Low | Low | High | High | Low | Unclear | Unclear | **High** |
| Gomez (2014) | Low | High | High | High | Low | Unclear | Unclear | **High** |
| Achenbach (1993) | High | High | High | High | Unclear | Unclear | Low | **High** |
| Beeghly (1995) | Unclear | High | High | High | Low | Unclear | Unclear | **High** |
| Holditch-Davis (2014) | Low | Unclear | High | High | Low | Low | Unclear | **High** |
| Melnyk (2001) | Unclear | Unclear | Unclear | Unclear | Unclear | Low | Unclear | **Unclear** |
| Nurcombe (1984) | High | High | High | High | Low | Low | Unclear | **High** |
| Pridham (2006) | Low | Unclear | High | High | Low | Unclear | Unclear | **High** |
| Melnyk (2004) | Unclear | Unclear | Unclear | Unclear | Unclear | Low | Unclear | **Unclear** |
| Berthelsen (2016) | High | High | High | High | Unclear | Unclear | Low | **High** |
| Eloniemi-Sulkava (2009) | Low | High | Low | High | High | Unclear | Low | **High** |
| Mittelman (1996) | High | High | High | High | High | High | High | **High** |
| Robinson (2007) | Low | Unclear | High | Unclear | Unclear | Unclear | Unclear | **High** |
| Blauw-Hospers (2011) | High | High | High | High | Unclear | Unclear | Low | **High** |
| Watson (2009) | High | High | High | Low | Unclear | unclear | Low | **High** |
| Souaibi (2016) | High | High | High | High | High | Unclear | High | **High** |
| Tantirangsee (2015) | Low | Low | High | High | Low | Low | Unclear | **High** |
| Mueser (2013) | High | High | High | Low | Unclear | unclear | Low | **High** |
| Garcia-Huidobro (2001) | High | High | High | High | Low | Low | Unclear | **High** |
| Martinez (2003) | Low | High | High | High | Low | High | Unclear | **High** |
| Mitchell (2009) | Low | High | High | High | Low | Low | Unclear | **High** |
| Skoog (2006) | High | High | High | High | High | Low | Low | **High** |
| Galvin (2011) | Low | Low | Low | High | Low | Unclear | Unclear | **High** |
| Nayeri (2014) | Low | High | High | High | Unclear | Low | Unclear | **High** |
| Li (2003) | Low | Low | High | High | High | Low | Unclear | **High** |
| Toye (2016) | Low | Low | Low | Low | Low | Unclear | Unclear | **Unclear** |
| Boltz (2014) | High | High | High | High | Low | Low | Low | **High** |
| Fortinsky (2002) | High | High | High | High | Low | Low | Unclear | **High** |
| Fortinsky (2009) | Low | Low | Low | Unclear | Low | Unclear | Unclear | **Unclear** |
| Gitlin (2006) | High | High | High | High | High | Low | Unclear | **High** |
| Boudreau (2013) | High | High | High | High | Low | Low | Unclear | **High** |
| Butz (2005) | High | High | High | Low | Unclear | Unclear | Low | **High** |
| Chen (2013) | Unclear | Unclear | High | High | Low | Low | Unclear | **High** |
| Grover (2011) | High | Low | High | High | Low | Low | Unclear | **High** |
| Bass (2013) | High | High | High | High | Unclear | Unclear | High | **High** |
| Mavandadi (2017) | Low | High | High | High | Unclear | Unclear | Unclear | **High** |
| Callahan (2006) | Low | Low | Low | Low | Low | Low | Unclear | **Unclear** |
| Wang (2015) | Low | Low | Unclear | Unclear | Low | Low | Unclear | **Unclear** |
| Rodgers (1999) | Low | Low | Low | Unclear | Unclear | Unclear | Unclear | **Unclear** |
| Rodriguez-Gonzalo (2015) | Low | High | Low | Low | Low | Unclear | Unclear | **High** |

*ROB: risk of bias

† Sorted by caregiver type, then participant population
