# Supplementary material for: Translating evidence to patient care through caregivers: a systematic review of caregiver-mediated interventions
Source: BMC Med. 2018 Jul 12;16:105. doi: 10.1186/s12916-018-1097-4 (PMC6042352; doi:10.1186/s12916-018-1097-4)
Supplement: Supplementary file 6 — Type and Effectiveness of Interventions for Primary Study Outcomes in Studies with Moderate or High Strength Evidence According to the GRADE Criteria. Main results restricted to those studies with a moderate or high strength of evidence according to the GRADE criteria. (DOCX 13 kb) [file 12916_2018_1097_MOESM6_ESM.docx]

Additional file 6. Type and Effectiveness of Interventions for Primary Study Outcomes in Studies with Moderate or High Strength Evidence According to the GRADE Criteria

| **Type of Engagement** | **Patient Outcomes** | **Caregiver Outcomes** | **Provider Outcomes** | **Health System Outcomes** |
| --- | --- | --- | --- | --- |
| *Inform-Activate-Collaborate* | 4⇑ 4⇔ | 7⇑ 2⇔ | --- | 1⇑1⇔ |
| *Inform-Activate* | 12⇑ 3⇔ | 7⇑3⇔ | 1⇑ | 1⇑1⇔ |
| *Inform-Collaborate* | --- | 1⇑ | 1⇑ | --- |
| *Activate-Collaborate* | --- | --- | --- | --- |

Numbers represent the number of studies reporting on each outcome.

⇑: statistically significant positive effect of intervention; ⇔: no statistically significant effect of intervention or mixed effect of intervention; ⇓: statistically significant harmful effect of intervention
